# A Review of CAC-717, a Disinfectant Containing Calcium Hydrogen Carbonate Mesoscopic Crystals

**DOI:** 10.3390/microorganisms13030507

**Published:** 2025-02-25

**Authors:** Akikazu Sakudo, Koichi Furusaki, Rumiko Onishi, Takashi Onodera, Yasuhiro Yoshikawa

**Affiliations:** 1 School of Veterinary Medicine, Okayama University of Science, Imabari 794-8555, Ehime, Japan; y-yoshikawa@ous.ac.jp; 2 Mineral Activation Technical Research Center, Omuta 836-0041, Fukuoka, Japan; 3 Santa Mineral Co., Ltd., Minato-ku 105-0013, Tokyo, Japan; 4 Environmental Science for Sustainable Development, The University of Tokyo, Bunkyo-ku 113-8657, Tokyo, Japan; atonode@g.ecc.u-tokyo.ac.jp; 5 Research Center for Food Safety, The University of Tokyo, Bunkyo-ku 113-8657, Tokyo, Japan; 6 Institute of Environmental Microbiology, Kyowa Kako Co., Ltd., Machida 194-0035, Tokyo, Japan

**Keywords:** Ca(HCO_3_)_2_, disinfectants, disinfection, inactivation, nanotechnology, prion

## Abstract

Recent studies on utilizing biological functions of natural substances that mimic the mesoscopic structures (nanoparticles of about 50 to 500 nm) found in plant growth points and coral skeletons have been reported. After the calcium hydrogen carbonate contained in materials derived from plants and coral are separated, the crystals of the mesoscopic structure can be reformed by applying a high voltage under a specific set of conditions. A suspension of these mesoscopic crystals in water (CAC-717) can be used as an effective disinfectant. CAC-717 exhibits universal virucidal activity against both enveloped and non-enveloped viruses as well as bactericidal and anti-prion activity. Moreover, in comparison to sodium hypochlorite, the potency of CAC-717 as a disinfectant is less susceptible to organic substances such as albumin. The disinfection activity of CAC-717 is maintained for at least 6 years and 4 months after storage at room temperature. CAC-717 is non-irritating and harmless to humans and animals, making it a promising biosafe disinfectant. This review explores the disinfection activity of CAC-717 as well as the potential and future uses of this material.

## 1. Introduction

Disinfectants play a crucial role in controlling infectious diseases and protecting public health [1,2]. As such, disinfectants are widely used in healthcare settings, food, and pharmaceutical industries, and for general hygiene purposes [3]. Various types of disinfectants exist, including alcohols, chlorine-containing compounds, and aldehydes, each with specific applications and efficacy against different microorganisms [4]. The importance of disinfectants was shown during the recent pandemic caused by severe acute respiratory syndrome coronavirus 2 (SARS-CoV-2), which highlighted the need to identify novel decontamination procedures [5]. Research is ongoing to develop safer and more effective disinfectants, with nanotechnology emerging as a promising field [6].

While conventional disinfectants such as chlorine are effective, they have limitations and can contribute to antimicrobial resistance [7,8]. New disinfection methods, including oxidizing agents such as chlorine dioxide, show promise in overcoming these challenges [9]. Natural antimicrobial substances are being explored as safe alternatives to chemical disinfectants, particularly for applications in the food industry [10]. The efficacy of disinfectants depends on various factors, including concentration, reaction time, and environmental conditions [11]. Effective disinfection practices are essential in healthcare settings to prevent nosocomial infections [12]. Ongoing research focuses on developing safe, effective, and environmentally friendly disinfectants to combat a wide range of pathogens [13,14]. In this regard, natural product compounds are also being explored as alternatives to chemical-based disinfectants [15].

The growth points of plants and the skeleton of coral are rich in mesoscopic structures made of natural calcium hydrogen carbonate [Ca(HCO_3_)_2_] [16,17]. We previously isolated the calcium hydrogen carbonate from a mixture of plants, such as chrysanthemums, roses, and bamboo bark, as well as coral, and then subjected the material to ultrasonic vibration [16,17]. It has been established that the mesoscopic structure is reformed upon subsequent exposure of the isolated material to far-infrared electromagnetic radiation while applying a high direct current (DC) voltage [16,17,18]. The resulting material, known as CAC-717, contains a mesoscopic structure of ~50–500 nm.

This review provides a comprehensive update on CAC-717 as a disinfectant, focusing on the latest research that investigates its broad-spectrum antimicrobial properties. Specifically, the unique characteristics, production, and applications of CAC-717 are discussed. Likely modes of action of CAC-717 as a disinfectant are also proposed. The authors believe CAC-717 to be a next-generation disinfectant with significant potential for food safety, veterinary medicine, agriculture, and medical disinfection.

## 2. Production Process for CAC-717

The synthesis of CAC-717, which is a suspension of mesoscopic calcium hydrogen carbonate crystals in water, as described in Japan patent No. 5778328 [18], involves the following procedures:

### 2.1. Preparation of Suspension (A)

To prepare Suspension (A), Material (A) was added to distilled water at a concentration of 12.5% (*w*/*v*) using the apparatus described in Japan Patent No. 5778328 [18]. Material (A) consists of a combination of two components, Material (A1) and Material (A2), prepared as follows:

Material (A1-1): A dried and pulverized mixture of 10% (*w*/*w*) Field Thistle (leaf, stem, and flower parts), 60% (*w*/*w*) Mugwort (leaf and stem parts), and 30% (*w*/*w*) *Farfugium japonicum* (leaf and stem parts).

Material (A1-2): A dried and powdered mixture of 20% (*w*/*w*) *Rosa multiflora* (leaf and flower parts), 10% (*w*/*w*) *Geum japonicum* (leaf and stem parts), and 70% (*w*/*w*) raspberry (leaf, stem, and flower parts).

Material (A1): A combination of equal weights of Material (A1-1) and Material (A1-2).

Material (A2): A dried and pulverized mixture consisting of 25% (*w*/*w*) Acer (leaf and stem parts), 25% (*w*/*w*) *Betula platyphylla* var. *japonica* (leaf, stem, and bark parts), and 50% (*w*/*w*) *Cryptomeria japonica* (leaf, stem, and bark parts).

Material (A1) and Material (A2) were mixed in a ratio of 1:3 to form Material (A), which was then added to the reaction vessel. The reaction vessel contained distilled water and an insulated conductor wire, which was immersed together with Material (A).

**Figure 1:**
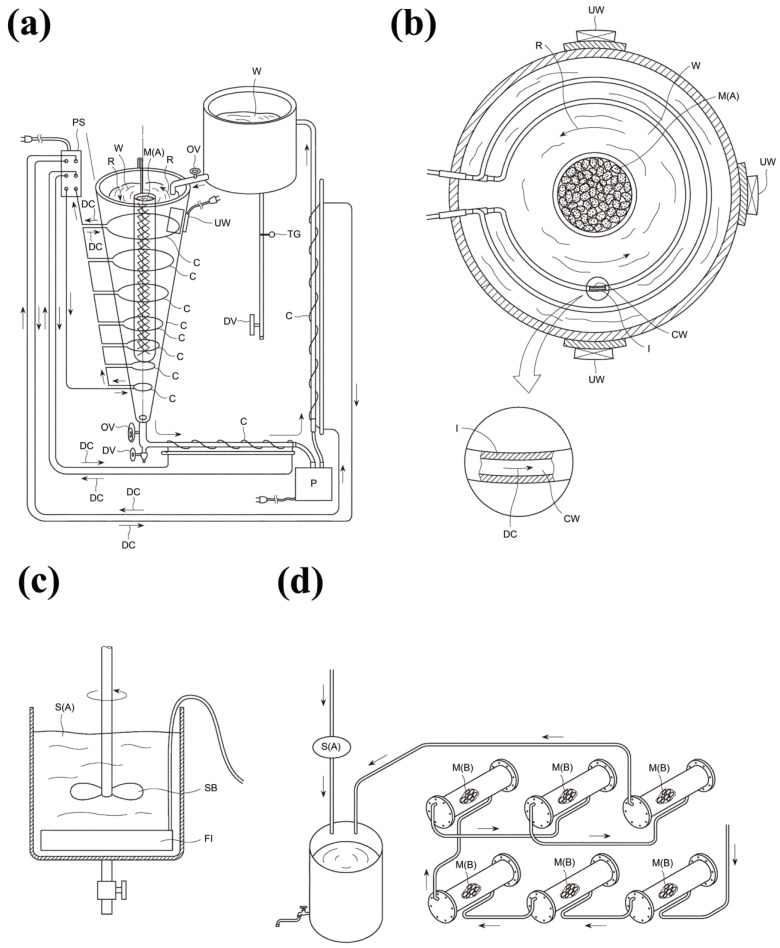
(**a**) Device for the production of Suspension (A). (**b**) Cross section of (**a**). (**c**) Far-infrared irradiation device. (**d**) Device for production of Suspension (B). W: water; M(A): material (A); I: insulator; CW: conductive wire; UW: ultrasonic wave generator; PS: power supply; DV: drain valve; TG: temperature gauge; C: conductive cable; SB: stirring blade; FI: far-infrared generator; S(A): suspension (A); M(B): mineralizing material (B); DC: direct current; P: power supply unit; R: water flow. Modified from Japan patent JP5778328B1 [18].

Then, a DC (8300 V, 100 mA) was applied to the conductor wire covered with a Teflon insulating layer (Figure 1a,b). The current generated a water flow around the conductor wire in the same direction as the DC to assist in the dissolution of the minerals. This water flow shows that a high-voltage DC generates a static electric field on an insulated surface of conductive wire, causing calcium ions from the plant material in the water flow to dissolve and combine with HCO_3_^−^ ions to form calcium hydrogen carbonate.

The system also includes a water circulation device driven by a pump that maintains the flow of water around the conductor wire to ensure uniform mixing. This continuous circulation promotes efficient extraction of minerals from Material (A) and maintains uniformity of the mineral suspension.

Next, ultrasonic vibrations are applied to the water to further enhance mineral dissolution. The ultrasonic generator uses a frequency of 50 kHz and an amplitude of 1.5/1000 mm to agitate the water molecules. This agitation accelerates the breakdown of Material (A). The consistent application of ultrasonic energy ensures high extraction efficiency.

Finally, the suspension is exposed to far-infrared radiation (Figure 1c). The far-infrared radiation, with wavelengths between 6 and 14 μm, enhances the dissolution of carbon dioxide in water. This process facilitates the formation of calcium hydrogen carbonate and transforms it into Suspension (A).

### 2.2. Preparation of Suspension (B)

To prepare Suspension (B), distilled water was passed through six water passage containers (Figure 1d). These containers were filled with different mineral imparting materials (B1 through B6) prepared as follows:

1. Material (B1): A mixture of 70% (*w/w*) limestone, 15% (*w/w*) fossil coral, and 15% (*w/w*) shell.
2. Material (B2): A mixture of 40% (*w/w*) limestone, 15% (*w/w*) fossil coral, 40% (*w/w*) shell, and 5% (*w/w*) activated carbon.
3. Material (B3): A mixture of 80% (*w/w*) limestone, 15% (*w/w*) fossil coral, and 5% (*w/w*) shell.
4. Material (B4): A blend of 90% (*w/w*) limestone, 5% (*w/w*) fossil coral, and 5% (*w/w*) shell.
5. Material (B5): A blend of 80% (*w/w*) limestone, 10% (*w/w*) fossil coral, and 10% (*w/w*) shell.
6. Material (B6): A mixture of 60% (*w/w*) limestone, 30% (*w/w*) fossil coral, and 10% (*w/w*) shell.

The shell used in these materials can be derived from *Haliotis* spp., *Sulculus diversicolor*, and *Balanus* spp. These materials (B1~B6) were placed in their respective water passage containers, with water flowing sequentially through each container, absorbing different minerals at each stage. Water was then passed sequentially through each container, beginning with Material (B1) and continuing through Material (B6). The specific compositions of the mineral imparting materials allowed different minerals to dissolve in the water at each stage, forming Suspension (B).

### 2.3. Synthesis of CAC-717

To synthesize CAC-717, Suspension (A) and Suspension (B) were mixed at a ratio of 1:10. This optimum ratio generates a CAC-717 of pH 12.3 containing 6.9 mM calcium hydrogen carbonate crystals with a mesoscopic structure. CAC-717 displays disinfectant activity and has been classified by the US Food and Drug Administration (FDA) as a Class 1 disinfectant (FDA/USA Regulation No. 880.6890 Class 1) [16]. CAC-717 is commercially available from Santa Mineral Co., Ltd. (Tokyo, Japan).

## 3. Inactivation of Bacteria by CAC-717

CAC-717 has been shown to be effective as a disinfectant against a wide range of bacteria as detailed in investigations that highlighted its rapid and potent bactericidal properties (Table 1).

### 3.1. Inactivation of Salmonella by CAC-717

*Salmonella enterica*, a significant pathogen causing foodborne illnesses, poses considerable public health risks, particularly in poultry and egg production [20,21,22]. CAC-717 has been evaluated for its efficacy using *Salmonella enterica* serovar Abony NCTC 6017 [19]. CAC-717 achieved a marked reduction in viable cell counts of *S.* Abony [19]. Initial concentrations of 2.14 × 10^7^ colony forming unit (CFU)/mL were reduced to 2.00 × 10^5^ CFU/mL within 1 min of treatment, and complete inactivation was achieved within 2 min (i.e., below detectable limits; less than 1 CFU/mL). The decimal reduction time (*D*-value) for *S. enterica*, which corresponds to the time needed to achieve a 90% reduction in viable bacteria, was determined to be 0.080 min for CAC-717. This *D*-value compares favorably to that of 4 ppm sodium hypochlorite (0.195 min (11.71 s)) [23], which was determined using the Association of Official Analytical Chemists (AOAC) Official method 960.09 [24]. The mechanism of action of CAC-717 was investigated. Polymerase chain reaction (PCR) analyses revealed that CAC-717 treatment reduced the integrity of bacterial DNA as evidenced by a progressive reduction in band intensity over time. Specifically, upon treatment with CAC-717 the DNA band intensity decreased to 44.46% at 1 min, then to 38.75% at 2 min, and was undetectable at 5 and 10 min.

### 3.2. Inactivation of Escherichia coli by CAC-717

Some serotypes of the Gram-negative bacterium *E. coli* are associated with outbreaks of food poisoning [25]. The nonpathogenic model *E. coli* HST04 strain (*dam*^−^*, dcm*^−^) was subjected to CAC-717 treatment [19].

The initial concentration of *E. coli* used in experiments was 1.52 × 10^9^ CFU/mL. CAC-717 treatment led to a substantial decrease in viable cell count (4.00 × 10^8^ CFU/mL) after 1 min and a further reduction (7.50 × 10^6^ CFU/mL) after 2 min [19]. Complete inactivation was observed at 5 min (i.e., below the detection limit; less than 10 CFU/mL). The *D*-value for *E. coli* was estimated to be 0.290 min, demonstrating potent and fast bactericidal action. Moreover, PCR analysis of *E. coli* treated with CAC-717 showed significant DNA damage. Band intensity analysis indicated a decrease from 100% at 0 min to 65.77% at 2 min, 31.38% at 5 min, and 4.13% at 10 min.

### 3.3. Inactivation of Xanthomonas campestris pv. Campestris (Xcc) by CAC-717

*Xcc* is a Gram-negative bacterium responsible for black rot in cruciferous crops [26,27,28]. Conventional disinfection procedures to control black rot, such as the use of sodium hypochlorite, are problematic due to environmental and safety concerns [28,29]. However, recent studies have demonstrated that CAC-717 offers an efficient approach to seed disinfection without compromising seed viability or growth [17].

The bactericidal activity of CAC-717 against *Xcc* was evaluated [17]. Firstly, an initial *Xcc* concentration of 8.22 log_10_ CFU/mL was subjected to CAC-717 treatment. The *D*-value for *Xcc* treated with CAC-717 was calculated to be 0.319 min, indicating rapid bacterial inactivation compared to conventional hot water treatment [30,31], which had a *D*-value of 2.137 min [17].

Next, to prepare *Xcc*-contaminated seeds, cabbage seeds were soaked in a suspension of *Xcc* (10 log_10_ CFU/mL) and dried. The *Xcc*-contaminated seeds were then suspended in either 1 mL of distilled water or CAC-717 for 30 min at 25 °C. Seeds were subsequently suspended in sterilized distilled water (500 µL) and the resultant supernatants plated onto a growth medium to determine the viable bacterial cell count. After a 30 min treatment with CAC-717 at 25 °C, the viable cell count in treated seeds was significantly reduced to 0.36 log_10_ CFU/mL, compared with 3.52 log_10_ CFU/mL observed in seeds treated with distilled water. Furthermore, the results from this study indicated that inactivation of *Xcc* by CAC-717 involved modifications to the bacterial genomic DNA.

The efficacy of CAC-717 was shown in reducing disease incidence in contaminated seeds. Incidence of black rot after 5 days of cultivation at 25 °C dropped to 26.67% ± 3.33% following CAC-717 treatment, a significant decrease compared to 56.67% ± 8.82% in the distilled water control group. Crucially, seed germination rates (90.00% ± 5.77%) and plant stem growth (28.27 mm ± 1.27 mm) were unaffected after CAC-717 treatment by comparison to the untreated control.

**Figure 2:**
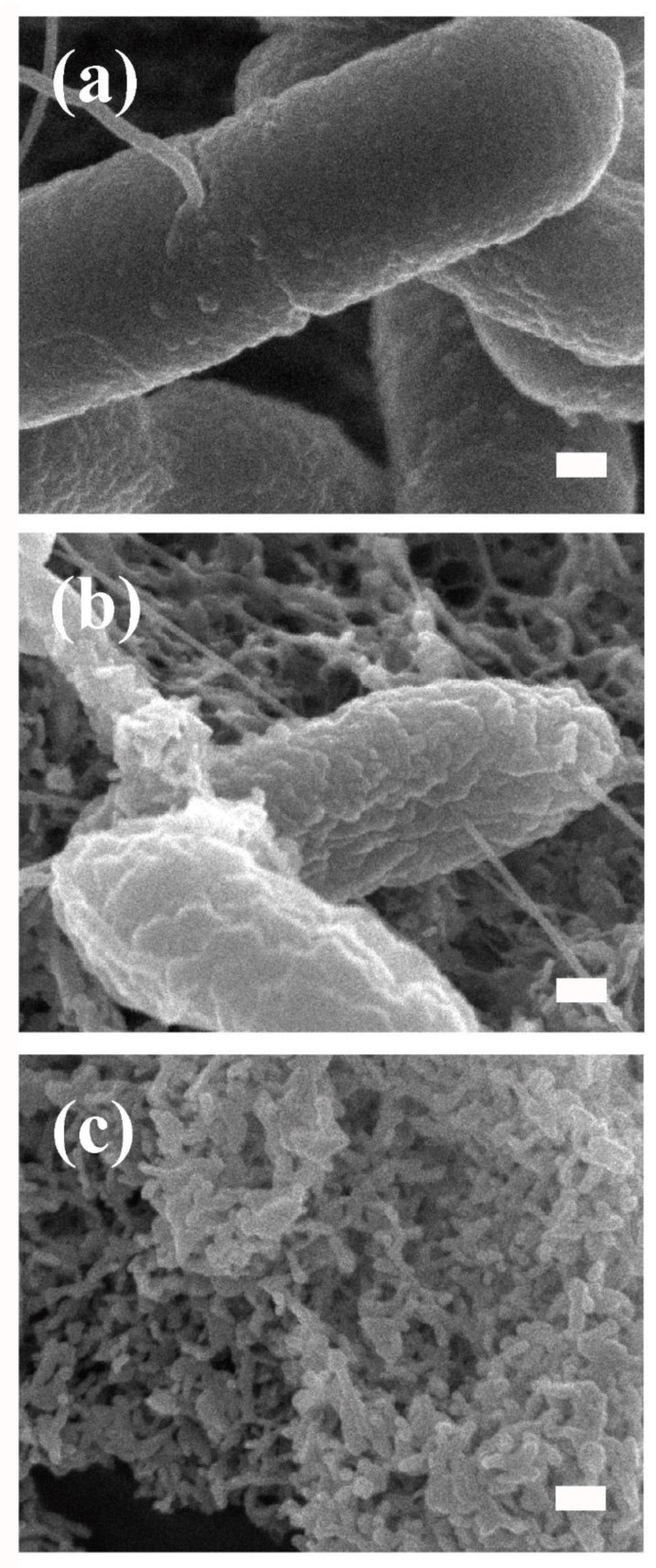
Changes in surface morphology of *Xcc* after CAC-717 treatment. A *Xcc* suspension, either untreated (**a**) or treated with an equal volume of CAC-717 for 0.5 min at 25 °C (**b**), and CAC-717 only (**c**) were fixed with glutaraldehyde. After treatment with osmium tetroxide, the samples were subjected to scanning electron microscopy (SEM) analysis (JSM-7500F; JEOL Ltd., Tokyo, Japan). Mesoscopic crystals are attached to the surface of *Xcc*. The scale bar corresponds to 100 nm.

In our additional preliminary experiments, scanning electron microscopy (SEM) was used to analyze the surface morphology of *Xcc* before and after CAC-717 treatment at 25 °C for 0.5 min (Figure 2a,b). SEM analysis showed that the surface of *Xcc* cells treated with CAC-717 for 0.5 min appeared to be slightly roughened and attached with nanoparticles. SEM analysis of the CAC-717 alone showed that the nanoparticles in CAC-717 were aggregated (Figure 2c). This aggregation of nanoparticles may be an artifact from the drying process.

In summary, CAC-717 was found to be an effective disinfectant for seeds contaminated with *Xcc*. However, there are several limitations with this study that need to be addressed. First, because only a single isolate of *Xcc* was tested, the broader applicability of CAC-717 against other *Xcc* isolates and various seed-borne bacterial species remains to be determined. Nonetheless, CAC-717 can inactivate other bacteria such as *Salmonella* and *E. coli*, suggesting its potential efficacy against a broader spectrum of bacterial pathogens. Second, the efficacy of CAC-717 as a treatment of seeds for other *Brassica oleracea* beyond cabbage remains to be evaluated. In order to fully assess the efficacy of CAC-717, further studies with a variety of *Xcc* isolates and different types of seed within the *Brassica oleracea* family are needed. In addition, the effect of temperature variation during disinfection treatment needs to be evaluated. Third, CAC-717 has been used to treat relatively small batches of seed. It remains unclear whether CAC-717 maintains its efficacy with larger seed lots. Thus, studies focusing on the relationship between the scale of seed treatment and inactivation efficacy, along with optimization of treatment duration and temperature, are essential in assessing the applicability of CAC-717 as a method for combating black rot.

It is also critical to compare the disinfection efficacy of CAC-717 with traditional seed treatments, such as hot water treatment at 50 °C [32], and to explore the potential benefits of combined treatment strategies. Indeed, important insights could be gained from such studies by assessing both disinfection efficacy and germination rates in seed contaminated with seed-borne bacteria. These comparative analyses will contribute to the development of more effective and reliable seed disinfection protocols.

Although CAC-717 is known to damage the genomic DNA of bacteria such as *E. coli* and *Salmonella*, the mechanism by which it inactivates *Xcc* remains unclear. One hypothesis is that CAC-717 disrupts bacterial membranes, exposing genomic DNA to degradation by DNase. However, it is uncertain whether DNA damage is a direct contributor to the bactericidal mechanism of CAC-717 or merely a secondary effect following cell death. Further mechanistic studies are required to better understand the bactericidal activity of CAC-717.

Taken together, these studies indicate that CAC-717 is an effective bactericidal agent against *Salmonella*, *E. coli*, and *Xcc*. Moreover, CAC-717 is a safe, non-toxic disinfectant that has potential applications in the food industry.

## 4. Inactivation of Viruses by CAC-717

CAC-717 has demonstrated significant virucidal effects across a wide range of enveloped and non-enveloped viruses (Table 2).

### 4.1. Inactivation of Influenza Viruses by CAC-717

The influenza virus, which primarily targets the respiratory tract and causes seasonal epidemics worldwide [36], can persist on surfaces for extended periods of time, posing a risk of transmission in healthcare settings [37]. Nakajima et al. [16] investigated the virucidal properties of CAC-717 against 5.5~6.5 log_10_ TCID_50_ (median tissue culture infectious dose) of influenza A virus (H3N2 subtype). This study quantitatively demonstrated that CAC-717 achieved 4~3 log_10_ reduction in virus titer (TCID_50_) within 1 min of treatment, with infectivity becoming undetectable (i.e., less than 1.5 TCID_50_) after 15 min at room temperature. Moreover, this rapid inactivation was unaffected by the presence of organic material (10% bovine serum albumin, BSA).

### 4.2. Inactivation of Feline Calicivirus by CAC-717

Noroviruses are the causative agents of foodborne gastroenteritis worldwide [38]. Because human noroviruses are difficult to culture, surrogates, such as murine norovirus (MNV) and feline calicivirus (FCV), are used for research purposes [39]. Sakudo et al. [19] extended the evaluation of CAC-717 to non-enveloped viruses, specifically focusing on FCV, a surrogate for human norovirus. Generally, non-enveloped viruses are more resistant to disinfectants than enveloped viruses [40]. Quantitative data indicated that CAC-717 treatment of FCV with an initial viral titer of 5.86 log_10_ TCID_50_/mL reduced the titer by 3 log_10_ TCID_50_ within 1 min and more than 4 log_10_ TCID_50_ within 2 min, thereafter reaching undetectable levels (less than 10 TCID_50_/mL). The mechanism of CAC-717 inactivation was investigated using real-time PCR analyses. This analysis showed a significant decrease in viral genome amplification following treatment with CAC-717. For example, FCV genomic RNA amplification was inhibited within 2 min of exposure to the disinfectant, reducing the viral titer below detectable levels. These observations suggest that CAC-717 promotes genetic instability.

### 4.3. Inactivation of Noroviruses by CAC-717

Shimakura et al. [33] provided insights into the efficacy of CAC-717 against human norovirus (HuNV) and its surrogate, MNV. This study reported a 3.25-log reduction in HuNV RNA after 30 min of CAC-717 treatment at reaction ratio of virus sample to CAC-717 of 1:1 with purified HuNV from a fecal specimen. For MNV, treatment with CAC-717 led to more than 4 log_10_ reduction after only 1 min at reaction ratio of 1:9. Transmission electron microscopy demonstrated capsid disruption of MNV, supporting the conclusion that CAC-717 directly damages the viral structure. This study also showed that the efficacy of CAC-717 in stool suspensions remained high, indicating a lesser susceptibility to organic substances compared to conventional disinfectants such as sodium hypochlorite. Indeed, the disinfection efficiency of 1000 ppm sodium hypochlorite is markedly reduced when exposed to more than 2.5% BSA. These findings suggest CAC-717 is suitable for use in environments containing organic material.

### 4.4. Inactivation of Severe Acute Respiratory Syndrome Coronavirus 2 and Other Viruses by CAC-717

Severe acute respiratory syndrome coronavirus 2 (SARS-CoV-2) [41], the virus responsible for coronavirus disease 2019 (COVID-19) [42], primarily targets the respiratory system, causing symptoms ranging from a mild upper respiratory infection to severe pneumonia and acute respiratory distress syndrome [43,44]. Yokoyama et al. [34] evaluated the effect of CAC-717 on SARS-CoV-2, including various strains such as alpha, beta, gamma, and delta. This study tested the virucidal effect of CAC-717 on SARS-CoV-2 at different reaction ratios of virus sample to CAC-717 of 1:9, 1:49, and 1:99 for 15, 30, 60, or 300 s at 20 °C. Using these conditions, SARS-CoV-2 isolates from SARS-CoV-2/WK-521 [45] as well as hCoV-19/Japan/QK002/2020 (alpha variant: EPI_ISL_804008), hCoV-19/Japan/TY7-501/2021 (beta variant: EPI_ISL_833366), hCoV-19/Japan/TY8-612/2021 (gamma variant: EPI_ISL_1123289), and SARS-CoV-2/KH-1, and SARS-CoV-2/KH-25/2021 (delta variants) distributed from the National Institute of Infectious Diseases, Tokyo, Japan [34] were all inactivated. CAC-717 achieved > 4 log_10_ reduction in the infectivity of SARS-CoV-2 (SARS-CoV-2/WK-521) within just 15 s at a 1:9 and 1:49 dilution ratio. The reduction remained consistent over longer exposure times of up to 5 min, maintaining more than a 4 log_10_ reduction. Inactivation activity against SARS-CoV-2 was also demonstrated using other SARS-CoV-2 strains (hCoV-19/Japan/QK002/2020, hCoV-19/Japan/TY7-501/2021, hCoV-19/Japan/TY8-612/2021, SARS-CoV-2/KH-1, and SARS-CoV-2/KH-25/2021) at a dilution ratio of 1:49 with a 4~3 log_10_ reduction after 5 min treatment in all cases.

The above inactivation effect of SARS-CoV-2 by CAC-717 revealed by Yokoyama et al. [34] was recently supported and extended by the results of a study by Kirisawa et al. [35]. Although inactivation efficiency of disinfectants can vary depending on the particular class of virus [46,47,48], CAC-717 was shown to inactivate all six types of animal viruses as follows: (i) enveloped double-stranded (ds) DNA viruses, including infectious bovine rhinotracheitis virus (IBRV), pseudorabies virus (PrV), canine herpesvirus 1 (CHV-1), and equine herpesvirus 1 (EHV-1); (ii) non-enveloped ds-DNA viruses, including bovine adenovirus 7 (BAdV-7); (iii) non-enveloped single-stranded (ss)-DNA viruses, including canine parvovirus 2 (CPV-2); and (iv) enveloped ssRNA viruses, including bovine parainfluenza virus 3 (BPIV-3), bovine respiratory syncytial virus (BRSV), canine distemper virus (CDV), Newcastle disease virus (NDV), vesicular stomatitis virus (VSV), SARS-CoV-2, bovine coronavirus (BCoV), swine influenza A virus (pdm09, H1N1) (SwIV), equine influenza A virus (H3N8) (EqIV), bovine viral diarrhea virus I (BVDV-I) and bovine viral diarrhea virus II (BVDV-II); (v) non-enveloped ssRNA viruses, including foot-and-mouth disease virus (FMDV) (type A, type O, and type Asia 1), bovine rhinitis B virus (BRBV), and FCV; and (vi) non-enveloped dsRNA viruses, including bovine rotavirus (BRoV) and bulbul ortho-reovirus (BuROV) by 4~3 log_10_ reduction (Table 2). In summary, these results demonstrate that CAC-717 has a broad spectrum of viral inactivation. Furthermore, CAC-717 was stored at room temperature in the dark for 6 years and 4 months, and its virucidal activity against IBRV remained unchanged. This indicates that CAC-717 maintains its inactivation activity even after long-term storage.

Taken together, these studies highlight the broad utility of CAC-717 as a disinfectant against a variety of viruses. According to the U.S. Environmental Protection Agency (USEPA) “Guide Standard and Protocol for Testing Microbiological Water Purifiers”, the minimum performance standards for virus inactivation efficiency are a 4-log reduction [49]. CAC-717 fulfills these criteria with a reported 4~3-log reduction in virus titer (Table 2).

## 5. Inactivation of Prions by CAC-717

Prion diseases, or transmissible spongiform encephalopathies, including Creutzfeldt-Jakob disease (CJD), Gerstmann–Sträussler–Scheinker syndrome (GSS), and fatal familial insomnia (FFI) in human, bovine spongiform encephalopathy (BSE) in cattle, scrapie in sheep and goats, are neurodegenerative disorders characterized by the accumulation of misfolded prion protein (PrP), an abnormal isoform of PrP (PrP^Sc^), in the central nervous system [50,51,52,53]. PrP^Sc^ is generated by the conformational change in the cellular isoform of PrP (PrP^C^) [54,55]. Prions are known to be the most resistant type of pathogen [52,56,57], posing significant challenges for inactivation in medical, veterinary, and industrial settings. The development of safe, effective, and non-corrosive disinfectants is essential for mitigating the risk of prion transmission. The effectiveness of CAC-717 against prions has been reported (Table 3).

**Figure 3:**
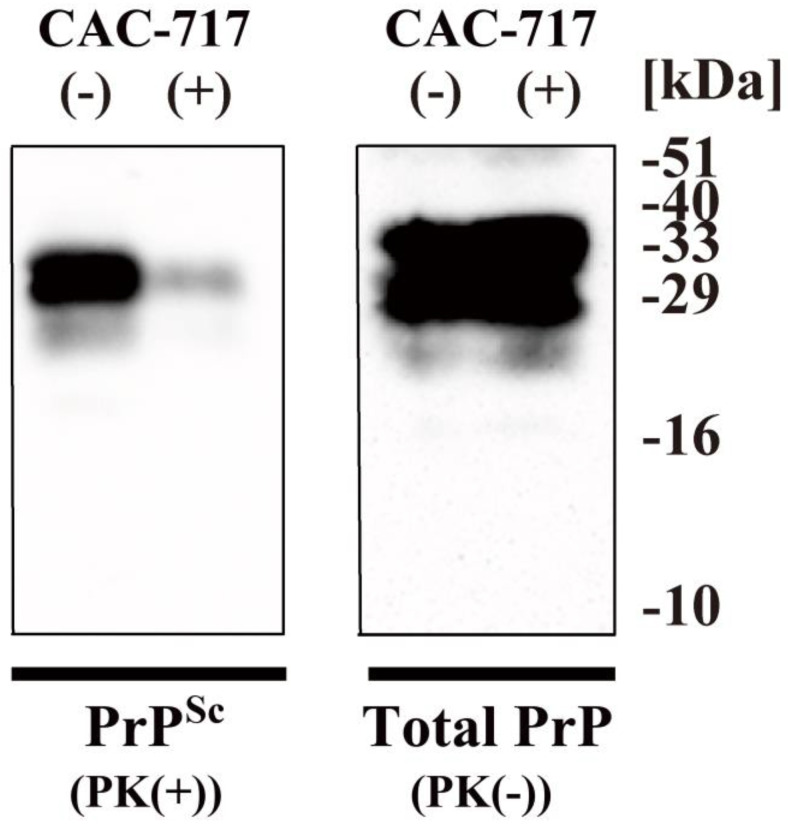
Degradation of prion in hamster brain homogenate by CAC-717 treatment. A homogenate of scrapie prion (263K)-infected hamster brain was diluted with phosphate-buffered saline (PBS) and subsequently mixed with an equal volume of CAC-717 (+) or PBS alone (−) as a negative control. Samples were incubated at 25 °C for 1 h and then subjected to either treatment with proteinase K (PK) to determine abnormal isoform of prion protein (PrP^Sc^) levels (PK(+)) or no treatment to determine the levels of total PrP (prion protein) (PK(−)). Aliquots of the samples were analyzed by sodium dodecyl sulfate–polyacrylamide gel electrophoresis (SDS-PAGE) followed by Western blotting using 3F4 anti-PrP antibody, which recognizes amino acid residues 107–112 of human PrP [61]. The level of PrP^Sc^ was reduced in 263K prion-infected hamster brain homogenate after treatment with CAC-717, but levels of total PrP were almost the same in CAC-717-treated and PBS-treated samples. Molecular mass markers (kDa) are indicated on the right-hand side of the blots.

PrP^Sc^ is known for its resistance to proteolytic degradation by proteinase K (PK) [56]. In experiments using PK, CAC-717-treated prions showed substantial susceptibility to digestion, with near-complete degradation in the presence of 4 μg/mL PK. Sakudo et al. [58] analyzed the effect of CAC-17 on cell lysates of Chandler scrapie prion-infected cells (ScN2a) using Western blotting. Whereas CAC-717-treated cell lysates with no PK digestion showed PrP signals, cell lysates treated with PK revealed an absence of PrP signals. These findings indicated that CAC-717 disrupts the structural integrity of PrP^Sc^, rendering it more amenable to enzymatic digestion. Here, to confirm this result, we performed additional preliminary experiments (Figure 3). Brain homogenates from hamsters infected with scrapie prion (strain 263K) were treated with CAC-717 at 25 °C for 1 h and then subjected to Western blotting. The amount of PrP^Sc^ was greatly reduced in CAC-717-treated brain homogenates after PK digestion compared to untreated brain homogenate. Interestingly, the total PrP (i.e., both PrP^C^ and PrP^Sc^) was almost unchanged between CAC-717-treated and non-treated samples. These findings are consistent with previous reports using ScN2a cell lysates [58], which show that CAC-717 does not degrade either PrP^C^ or PrP^Sc^. Rather, CAC-717 enhances PK sensitivity by presumably altering the conformation of PrP.

Further confirmation of the prion-inactivating potential of CAC-717 was provided by conducting a series of bioassays in mice [58]. Four among six Tga20 mice (PrP-overexpressing mice) [60] intracerebrally inoculated with Chandler prions treated with CAC-717 survived up to the time of study termination (368 days post inoculation), whereas all five mice inoculated with phosphate-buffered saline (PBS)-treated ScN2a cell lysate succumbed to the disease within 140.8 ± 11.9 days.

Protein misfolding cyclic amplification (PMCA) assays further supported this mechanism. The PMCA analysis revealed that CAC-717-treated lysates showed a marked decrease in prion seeding activity [58]. The PMCA_50_ value (median dose of PMCA) for untreated samples was recorded at log_10_9.95, while CAC-717 treatment reduced this to log_10_5.20, reflecting a 4.75-log reduction in seeding potential. This finding suggests that CAC-717 not only degrades prion proteins but also impairs their ability to seed new PrP^Sc^ formation.

Iwamaru et al. [59] tested the use of CAC-717 ceramic (CAC-717 absorbed on ceramic) for inactivating prions in Chandler strain scrapie-infected brain homogenates (ScBH). When treated once with 30% (*w*/*v*) CAC-717 ceramic for 30 min, the prion seeding activity, measured by PMCA, showed a 3.0-log_10_ reduction in PMCA_50_/mg. A double treatment of CAC-717 increased the reduction to 4.2-log PMCA_50_/mg. This two-step treatment approach significantly accelerated prion inactivation. Moreover, the addition of sodium dodecyl sulfate (SDS) to the CAC-717 ceramic treatment enhanced prion inactivation still further. This synergistic effect was quantifiable as more than a 6.0-log reduction in prion seeding activity in PMCA, comparable to the performance of 1 M NaOH, which is a conventional but highly corrosive disinfectant.

This study also incorporated a mouse bioassay to assess the effect CAC-717 ceramic treatment on in vivo prion infectivity. Tga20 mice intracerebrally inoculated with untreated ScBH succumbed to prion disease within an average of 67 ± 2 days. In contrast, mice inoculated with ScBH treated once with CAC-717 ceramic had an extended mean survival time of 112 ± 20 days. For ScBH treated twice with CAC-717 ceramic, survival times were further prolonged to 119 ± 21 days. Treatment with CAC-717 ceramic combined with SDS treatment resulted in the complete inactivation of prion with no disease development observed in any of the inoculated mice up to 365 days post-inoculation. These findings demonstrate the progressive efficacy of repeated CAC-717 and SDS combination treatment to inactivate prions.

Taken together, recent studies have highlighted the potential of CAC-717 for prion inactivation. To fully establish CAC-717 as a reliable prion disinfectant, future research should focus on comparative studies involving different prion strains. Furthermore, diverse environmental conditions in various biological matrices, such as blood or tissue, needs further exploration. The potential for synergistic effects with other disinfectants or processing methods should also be explored. Understanding the precise molecular interactions and structural changes induced by CAC-717 will further elucidate its mechanism of action and inform the development and refinement of new disinfectants based on its properties.

## 6. Hypothetical Disinfection Mechanisms of CAC-717

**Figure 4:**
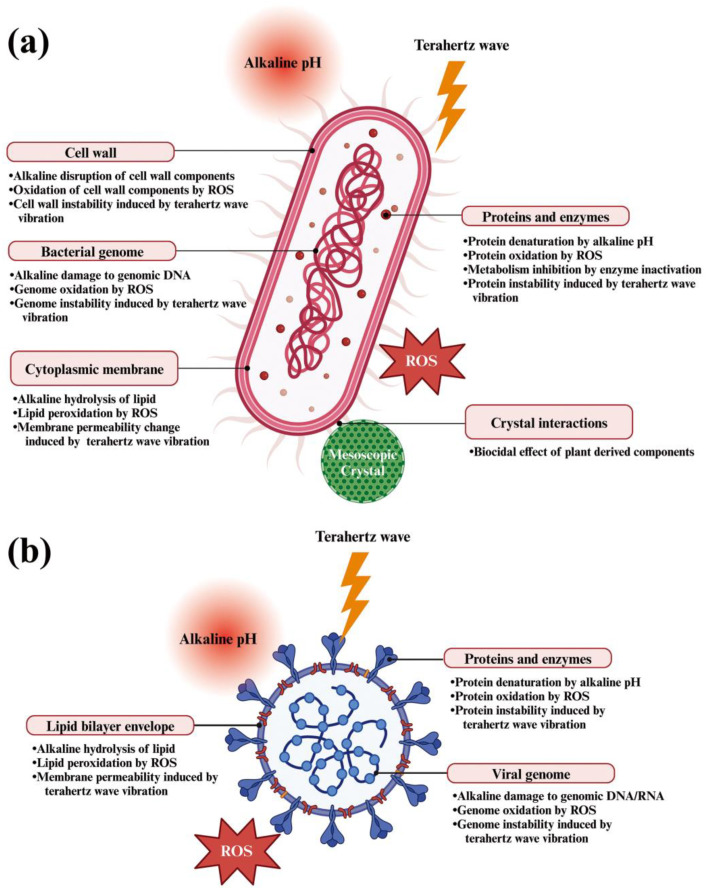
A schematic of the hypothetical action of CAC-717 on microorganisms including bacteria (**a**) and viruses (**b**). Created in BioRender. Sakudo, A. (2025) https://BioRender.com/u73v812 and https://BioRender.com/x48x567 (accessed on 4 February 2025).

Although compelling evidence that CAC-717 inactivates microorganisms has been accumulated, the specific mechanisms by which CAC-717 achieves microbial inactivation remain unclear and warrant further exploration. In this section, we speculate on potential multiple mechanisms of disinfection mediated by CAC-717 (Figure 4).

Circulating electrons in the core of mesoscopic crystals emit electron pulses (terahertz waves) [16]. Terahertz waves cause molecular vibrations in biopolymers such as lipids, sugars, proteins, and nucleic acids, resulting in structural changes [62]. These structural changes may affect the function and stability of biomolecules, potentially interfering with cellular physiological processes. Water molecules within viral capsids and bacterial cells also resonate at these wavelengths [63], leading to alterations in hydrogen bonding networks and water cluster dynamics, which may influence membrane permeability and biochemical reactions that could inactivate the pathogens.

The high pH of CAC-717 with a pH of 12.3 [16] may also explain, at least in part, its bactericidal activity by inducing alkaline hydrolysis, promoting the breakdown of outer proteins as well as peptidoglycan in cell walls, thereby leading to cell weakening and eventual lysis. The high pH of CAC-717 further inhibits key bacterial enzymes involved in metabolism and DNA replication, impeding energy production and biosynthesis, and rendering cells unable to divide while increasing their susceptibility to genomic DNA damage. However, Shimakura et al. [33] reported that although CAC-717 successfully inactivated human norovirus no such inactivation was observed using PBS with a pH of 12.6. This finding suggests that an alkaline pH alone is insufficient to explain the inactivation process, at least for human norovirus. Therefore, the biocidal activity of CAC-717 appears to involve mechanisms beyond simple alkaline hydrolysis.

Experiments using bacteria such as *E. coli* and *Salmonella* demonstrated that CAC-717 treatment caused the loss of PCR amplification of genomic DNA, suggesting modification and/or degradation of bacterial genomic DNA [19]. However, it should be noted that this does not necessarily indicate that nucleic acids are the primary target of action in the inactivation of bacteria. Instead, cell membrane disruption is likely to occur prior to any DNA damage.

Moreover, reactive oxygen species (ROS) are generated from particles of CaO, MgO, and ZnO as shown by multi-parameter flow cytometry [64]. In particular, calcium oxide-based nanoparticles, such as bioshell calcium oxide (BiSCaO), display potent microbiocidal activity [65]. The microbiocidal activity of BiSCaO is thought to be attributable to its high alkalinity and potential to generate ROS [66]. Likewise, CAC-717 may generate ROS from calcium hydrogen carbonate mesoscopic crystals under a specific set of conditions, similar to other disinfectants [67,68]. The potential generation of ROS from CAC-717 could induce lipid peroxidation, protein oxidation, as well as DNA damage. Cell death would result from compromised membrane integrity and/or cellular disfunction. In addition, we know that CAC-717 contains total organic carbon (TOC), which is likely derived from plant extracts and may include terpenes and polyphenols. These minor components may also contribute to the biocidal activity of CAC-717. Further studies on the disinfection activity of similar samples prepared in the absence of plant-derived materials would be interesting in evaluating the importance of these minor components in CAC-717.

For viruses, CAC-717 disrupts lipid envelopes and capsids through alkaline hydrolysis, disintegrating the viral structure and preventing the virus from infecting host cells [40,69]. The ROS-mediated lipid peroxidation of the envelope as well as protein oxidation further weakens the viral structure [70]. Essential viral proteins, such as those necessary for cell binding and replication, are susceptible to denaturation in this highly alkaline environment, inhibiting both the infection and replication processes [71,72]. The nucleic acid of the virus genome, including DNA and RNA, can be fragmented and/or modified, resulting in loss of infectivity.

Prions can be inactivated by CAC-717 through multiple mechanisms. The high pH induces denaturation, unfolding PrP and preventing the formation of a pathogenic conformation [52]. Because prions bind to the soil mineral montmorillonite [73], the mesoscopic crystals in CAC-717 might further interact with prions by adsorbing them and/or inducing structural changes that reduce infectivity or alter the effectiveness of inactivation treatments.

## 7. Practical Considerations and Applications of CAC-717

Chlorine-based disinfectants, such as sodium hypochlorite, while effective, pose safety and environmental risks [74]. For example, sodium hypochlorite can emit toxic gasses and cause metal corrosion. CAC-717, in contrast, has a pH of approximately 12.3 but becomes neutral around 8.84 upon contact with biological tissue [16]. Even after neutralization, CAC-717 maintains its anti-microbial activity. This observation indicates that its inactivation mechanism is not dependent on pH alone. Data in Japan patent No. 5778328 [18] showed no inflammatory response in human skin upon contact with CAC-717. CAC-717 was thoroughly applied to the skin of the upper arm, and the condition of the skin was observed 6 h later. No occurrence of contact dermatitis or atopic dermatitis was identified. Notably, while skin contact with the raw materials of CAC-717, such as plants from the Rosaceae and Asteraceae families, may cause skin inflammation, no inflammatory response was observed upon exposure to CAC-717 itself. The non-irritating profile of CAC-717 is important for potential applications of this material in the healthcare and food industry, where effective yet safe disinfectants are needed.

In medicine, CAC-717 shows substantial potential as an effective disinfectant with applications that support patient safety and improve clinical settings. Healthcare facilities, including hospitals and clinics, face ongoing challenges in infection control. The broad-spectrum antimicrobial efficacy of CAC-717 against both bacteria and viruses as well as prions is ideal for these environments. Its application on frequently touched surfaces such as bed rails, medical charts, workstations and door handles can significantly reduce pathogen transmission, thereby enhancing the overall safety of healthcare settings [75,76]. Moreover, CAC-717 can be utilized to disinfect reusable medical equipment, including surgical instruments and diagnostic devices, ensuring their sterility and minimizing the risk of post-operative infections or complications related to medical procedures [77,78]. The use of CAC-717 for routine surface disinfection has demonstrated a notable reduction in the risk of nosocomial and iatrogenic infection transmission [77,78]. Additionally, the potential for air disinfection within hospitals and clinics offers a proactive approach to mitigating airborne disease transmission [79,80]. The application of CAC-717 through methods such as spraying, misting, or atomization enhances comprehensive infection control strategies [81,82], providing an added layer of protection that complements standard disinfection protocols.

In public health contexts, CAC-717 can be utilized during outbreaks to control the spread of infectious diseases. Disinfecting public transport vehicles such as buses, trains, and stations, along with public buildings like schools and offices, helps lower transmission rates and protect communities [83]. Routine disinfection practices using CAC-717 in high-traffic areas could act as a proactive measure to enhance public safety.

In veterinary medicine, the development and application of effective disinfectants are critical to ensure biosecurity, control outbreaks, and maintain high standards of hygiene [84]. In veterinary clinics and animal care facilities, bacterial pathogens such as *Salmonella*, *E. coli*, *Clostridium difficile*, and methicillin-resistant *Staphylococcus aureus* (MRSA) contribute to significant health risks for both animals and veterinary staff [85]. CAC-717 can be effectively incorporated into daily disinfection routines within veterinary hospitals, particularly in areas where pathogen load is high, such as surgical suites, examination rooms, and kennels. CAC-717 could also be used to reduce the risk of zoonotic infections, preventing the spread of pathogens from animals to humans and vice versa. One of the practical benefits of CAC-717 is its non-toxic nature [16]. This feature is particularly advantageous for use in large animal facilities and farms, where frequent disinfection is necessary to control the spread of infectious agents. CAC-717 can also be applied during outbreaks of highly contagious diseases, such as foot-and-mouth disease or avian influenza, supporting its utility in emergency response protocols [35]. Using CAC-717 in combination with other biosecurity measures can enhance the overall effectiveness of containment efforts. Moreover, the integration of CAC-717 into disease surveillance by veterinary epidemiologists could enhance biosecurity during sample collection and transportation [86]. Thus, given its broad-spectrum efficacy, CAC-717 could be used as part of a comprehensive disinfection strategy to support animal health and prevent cross-species transmission of pathogens.

In the agricultural sector, CAC-717 has been proven to be beneficial for protecting crops from harmful pathogens [17], preventing the spread of disease, and supporting sustainable farming and high crop yields. Treating seeds with CAC-717 can block the transmission of infectious plant diseases to the soil, which is crucial for early-stage crop health [17]. Crop spraying with this disinfectant could help manage bacterial and viral plant diseases, ensuring that crops grow robustly and with minimal loss, thereby promoting a more stable and high-quality food supply.

In the food industry, the use of CAC-717 as a disinfectant will contribute to preventing contamination and ensuring food safety. Regular disinfection of food processing surfaces and equipment helps to minimize the risk of foodborne illnesses, maintaining a safer production environment [87]. Because CAC-717 is safe for humans, it can also be directly applied to washed fruits and vegetables, significantly reducing microbial loads and extending the expiration date of food products, which is crucial for both retailers and consumers in minimizing food waste.

## 8. Conclusions and Future Perspectives

The inactivation spectrum of CAC-717 covers almost the entire resistance hierarchy of microorganisms. However, there have been no reports on the effect of CAC-717 treatment on plant viruses or protozoa, including protozoan oocysts and cysts, as well as bacterial spores. In particular, bacterial spores play a crucial role in sterilization assurance level (SAL) due to their exceptional resistance to environmental stress and disinfectants [88]. The structure and chemical composition of bacterial spores contribute to their unique resistance properties, necessitating specialized approaches for inactivation [89]. Furthermore, biological indicators, often utilizing bacterial spores, are essential for validating sterilization processes across various industries [90].

Further studies to determine the effectiveness of CAC-717 on multi-drug-resistant microorganisms such as MRSA and Carbapenem-resistant Enterobacterales are required. The disinfection potential of CAC-717 is particularly promising for combating bacteria that exhibit resistance to multiple antimicrobial agents. The rise in drug-resistant pathogens has become an acute public health concern. Both the World Health Organization [91] and the US Centers for Disease Control and Prevention [92] have highlighted this trend as a critical threat to global health. Furthermore, the extensive and often indiscriminate use of antibiotics has likely influenced the environmental microbiome, facilitating the spread of drug-resistant bacterial strains [93]. This situation highlights the urgent need for novel and effective disinfection methods to eradicate multidrug-resistant bacteria, particularly within food production and processing environments. CAC-717 holds significant promise due to its bactericidal mechanism, which is believed to be equally effective against both multidrug-resistant and susceptible bacterial strains, ensuring comprehensive disinfection capabilities.

To date, research on prion inactivation by CAC-717 has been limited to scrapie prions, specifically the Chandler strain. However, it is important to recognize that prions exist in various forms in both humans and animals, each exhibiting distinct characteristics [50]. The resistance of prions to inactivation varies significantly depending on their species of origin. Consequently, extrapolating results from rodent-passaged prion strains to those affecting humans or bovines may not yield reliable conclusions. Even when human-derived prion strains are incorporated into experimental models, the data remain incomplete due to the diverse array of prion diseases, each producing different prion subtypes. The level of resistance can vary according to the specific nature of the prion involved (e.g., variant CJD, sporadic CJD, GSS, or FFI). Within sporadic CJD alone, type 1 and type 2 PrP^Sc^ proteins exhibit unique biochemical characteristics [94]. Additionally, sporadic CJD can manifest through six different genotype/PrP^Sc^ combinations (MM1, MM2, MV1, MV2, VV1, and VV2), resulting in five major sporadic CJD strains: MM1/MV1, MV2/VV2, MM2 cortical (MM2c), MM2 thalamic (MM2t), and VV1 [95,96]. Such variations probably influence the resistance of each of these prions [97,98]. Given this complexity, further investigation into the efficacy of CAC-717 across all prion types is essential. This includes conducting experiments with clinically derived prion strains to establish a robust understanding of its inactivation potential. Furthermore, recent reports suggest that the causative agents of other neurodegenerative diseases, such as Alzheimer’s disease, may operate via prion-like mechanisms of protein misfolding and propagation as a type of prion or prion-like factor (prionoid) [99,100,101,102,103,104]. Studies have shown that amyloid-β (Aβ) and tau proteins can self-propagate and spread between cells in a manner similar to prions [104,105,106]. These findings have raised concerns about the potential transmissibility of such proteins. Our recent study showed that CAC-717 mediated the degradation and/or dissociation of Aβ aggregates as well as inhibiting their formation [107]. Further studies are required to fully assess whether CAC-717 is suitable for practical applications in the decontamination of prion and prionoids.

Investigating the effect of CAC-717 on these pathogens would potentially help expand the applicability of this technology. Future research should focus on the practical use of disinfectants in different environments, particularly healthcare and animal facilities, as well as the agricultural and food industry. Conducting in-use testing can confirm the effectiveness of disinfectants under real-world conditions, ensuring that manufacturers’ guidelines meet the practical challenges. Such studies are essential for improving infection control protocols. To date, experiments using CAC-717 have been performed exclusively at room temperature. However, evaluating the effectiveness of CAC-717 under various temperature and humidity conditions as well as different organic loads is important. The results from such studies will define the suitable range of conditions for the use of CAC-717, potentially expanding its applications.

In terms of environmental considerations, CAC-717 is expected to be fully biodegradable. However, more data on its degradation efficiency and byproduct footprint in the environment is required [108]. Further research is needed to confirm how CAC-717 breaks down under various environmental conditions and whether it forms any persistent or harmful substances. Indeed, it is pertinent to compare the long-term environmental footprint of CAC-717 with that of conventional disinfectants [109]. Furthermore, there is a paucity of direct data on the effects of CAC-717 on ecosystems. This balance of efficacy, safety, and environmental considerations, including risk assessments, necessitate further studies. Specifically, the studies should aim to fully evaluate the environmental impact of CAC-717.

In addition to conducting a risk assessment of the environmental issues outlined above, toxicological profiling will be essential in confirming the safety of CAC-717. Although previous studies have indicated no adverse effects to human skin upon exposure to CAC-717 [18], toxicological studies to determine cytotoxicity in various mammalian cells are needed for a risk assessment. Furthermore, repeated and prolonged exposure to a suspension of CAC-717 as well as potential inhalation toxicity by exposure to an aerosolized or misted form of CAC-717 are also required. Comparative risk assessments with conventional disinfectants, such as hypochlorous acid and ethanol, will help define suitable applications for CAC-717. Moreover, although CAC-717 has been shown to effectively inactivate a broad range of pathogens including bacteria, viruses, and prions, it remains unclear whether the long-term use of CAC-717 can induce the emergence of microbial resistance against CAC-717 itself. As such, long-term surveillance studies will need to be conducted.

To date, the comprehensive structure of mesoscopic crystals in CAC-717 remains unclear. In broad terms, these mesoscopic crystals are thought to comprise a calcium carbonate (CaCO_3_) core surrounded by a surface layer of hydrated calcium hydrogen carbonate [Ca(HCO_3_)_2_]. This proposal is, at least in part, consistent with studies that found nanosized CaCO_3_ particles possess several defective sites, which exhibit significant hydrolytic activity and basic properties [110,111]. These findings may explain the observed ability of such particles to inactivate microorganisms [112,113]. Furthermore, it should be noted that the application of high DC voltage and/or far-infrared irradiation may influence the structure and porosity of the mesoscopic crystals. Optimization of these parameters could increase the microbiocidal activity of CAC-717. Data from specific measurements of surface area, porosity, and pore volume of mesoscopic crystals in CAC-717 are not currently available. Further chemical characterization of the precise stoichiometry of the CAC-717 is also needed.

The current production protocol yields 1000 L of CAC-717 from approximately 40–50 kg of plant material and 10–20 kg of coral and shells. To enable large-scale production, improvements in the manufacturing process are required to reduce costs. Currently, CAC-717 is produced using calcium hydrogen carbonate derived from plants, coral, and shells. To lower costs, the development of recovery technologies utilizing waste materials, such as food processing byproducts and fishery waste, might be considered. Additionally, the use of cheaper and more abundant carbonate minerals should be explored for the large-scale production of calcium hydrogen carbonate. Furthermore, enhancing the efficiency of raw material processing, optimizing raw material selection, refinement of the manufacturing process, and introduction of automation may collectively contribute to reducing production costs and facilitating large-scale production. These advancements would improve the feasibility of scaling up manufacturing for various applications, including medical, public health, veterinary, agricultural, and food industry uses.

Finally, it should be noted that the CAC-717 ceramic displays distinct advantages due to its innovative formulation. The mesoscopic structure of crystals deposited on ceramic substrates [59] can be stored in a solid state, allowing convenient preparation of the disinfectant by simply suspending the material in water as needed. Indeed, this feature will facilitate the rapid, large-scale production and deployment of the disinfectant. In conclusion, CAC-717 and CAC-717 ceramic offers efficient alternatives to conventional disinfectants such as hypochlorous acid and ethanol.

## Figures and Tables

**Table 1:** Inactivation of bacteria by CAC-717.

Microorganism (Strain or Type)	Classification	Sample Type	Reaction Volume Ratio (Sample: CAC-717), Exposure Time	Inactivation Effect	Evaluation Method	References
*Salmonella enterica* subsp. *enterica* serovar Abony (NCTC6017)	Gram-negative bacteria/animal pathogen	Bacterial suspension	1:1, 1 min	2-log reduction	Colony count assay	[19]
*Escherichia coli* (HST04 strain)	Gram-negative bacteria/non-pathogenic microorganism	Bacterial suspension	1:1, 2 min	2-log reduction	Colony count assay	[19]
*Xanthomonas campestris* pv. *campestris* (NGM120310-14)	Gram-negative bacteria/plant pathogen	Bacterial suspension	1:1, 1 min	3-log reduction	Colony count assay	[17]

For clarity, decimal places in the numbers of log_10_ reduction have been omitted.

**Table 2:** Inactivation of viruses by CAC-717.

Microorganism (Strain or Type)	Classification	Sample Type	Reaction Volume Ratio (Sample: CAC-717), Exposure Time	Inactivation Effect	Evaluation Method	References
Human influenza A virus (A/Aichi/2/68 (H3N2))	Enveloped ss-RNA virus/human pathogen	Virus-infected cell culture suspension	1:9, 1 min	4-log reduction	TCID_50_ assay	[16]
Swine influenza A virus (A/Swine/Wadayama/5/69 (H3N2))	Enveloped ss-RNA virus/human pathogen	Virus-infected cell culture suspension	1:9, 1 min	3-log reduction	TCID_50_ assay	[16]
Feline calicivirus (F9)	Non-enveloped ss-RNA virus/animal pathogen	Virus-infected cell lysate	1:1, 1 min	3-log reduction	TCID_50_ assay	[19]
Mouse norovirus (S7)	Non-enveloped ss-RNA virus/animal pathogen	Virus suspension	1:9, 1 min	4-log reduction	TCID_50_ assay	[33]
Human norovirus (derived from patients: GII.4 Sydney 2012)	Non-enveloped ss-RNA virus/human pathogen	Virus suspension	1:1, 30 min	3-log reduction	RT-qPCR combined with PMA binding assay	[33]
SARS-CoV-2 (hCoV-19/Japan/QK002/2020)	Enveloped ss-RNA virus/human pathogen	Virus-infected cell culture suspension	1:49, 5 min	3-log reduction	TCID_50_ assay	[34]
SARS-CoV-2 (hCoV-19/Japan/TY7-501/2021)	Enveloped ss-RNA virus/human pathogen	Virus-infected cell culture suspension	1:49, 5 min	3-log reduction	TCID_50_ assay	[34]
SARS-CoV-2 (hCoV-19/Japan/TY8-612/2021)	Enveloped ss-RNA virus/human pathogen	Virus-infected cell culture suspension	1:49, 5 min	4-log reduction	TCID_50_ assay	[34]
SARS-CoV-2 (SARS-CoV-2/KH-1/2021)	Enveloped ss-RNA virus/human pathogen	Virus-infected cell culture suspension	1:49, 5 min	4-log reduction	TCID_50_ assay	[34]
SARS-CoV-2 (SARS-CoV-2/KH-25/2021)	Enveloped ss-RNA virus/human pathogen	Virus-infected cell culture suspension	1:49, 5 min	4-log reduction	TCID_50_ assay	[34]
SARS-CoV-2 (SARS-CoV-2/WK-521)	Enveloped ss-RNA virus/human pathogen	Virus-infected cell culture suspension	1:9, 15 s	4-log reduction	TCID_50_ assay	[34]
SARS-CoV-2 (SARS-CoV-2/WK-521)	Enveloped ss-RNA virus/human pathogen	Virus-infected cell culture suspension	1:49, 15 s	4-log reduction	TCID_50_ assay	[34]
SARS-CoV-2 (SARS-CoV-2/WK-521)	Enveloped ss-RNA virus/human pathogen	Virus-infected cell culture suspension	1:49, 5 min	4-log reduction	TCID_50_ assay	[34]
Infectious bovine rhinotracheitis virus (Los Angeles)	Enveloped ds-DNA virus/animal pathogen	Virus suspension	1:9, 2 s	4-log reduction	TCID_50_ assay	[35]
Pseudorabies virus (MY-1)	Enveloped ds-DNA virus/animal pathogen	Virus suspension	1:9, 2 s	4-log reduction	TCID_50_ assay	[35]
Canine herpesvirus 1 (GCH-1)	Enveloped ds-DNA virus/animal pathogen	Virus suspension	1:9, 2 s	3-log reduction	TCID_50_ assay	[35]
Equine herpesvirus 1 (HH1)	Enveloped ds-DNA virus/animal pathogen	Virus suspension	1:9, 2 s	4-log reduction	TCID_50_ assay	[35]
Bovine adenovirus 7 (Fukuroi)	Non-enveloped ds-DNA virus/animal pathogen	Virus suspension	1:9, 2 s	4-log reduction	TCID_50_ assay	[35]
Canine parvovirus 2 (97-008)	Non-enveloped ss-DNA virus/animal pathogen	Virus suspension	1:9, 10 s	3-log reduction	TCID_50_ assay	[35]
Bovine parainfluenza virus 3 (BN-1)	Enveloped ss-RNA virus/animal pathogen	Virus suspension	1:9, 2 s	4-log reduction	TCID_50_ assay	[35]
Bovine respiratory syncytial virus (rs-52)	Enveloped ss-RNA virus/animal pathogen	Virus suspension	1:9, 2 s	3-log reduction	TCID_50_ assay	[35]
Canine distemper virus (KDK-1)	Enveloped ss-RNA virus/animal pathogen	Virus suspension	1:9, 2 s	3-log reduction	TCID_50_ assay	[35]
Newcastle disease virus (Miyadera)	Enveloped ss-RNA virus/animal pathogen	Virus suspension	1:9, 15 min	3-log reduction	TCID_50_ assay	[35]
Vesicular stomatitis virus (New Jersey)	Enveloped ss-RNA virus/animal pathogen	Virus suspension	1:9, 15 min	4-log reduction	TCID_50_ assay	[35]
SARS-CoV-2 (JPN/TY/WK-521)	Enveloped ss-RNA virus/human pathogen	Virus suspension	1:9, 2 s	4-log reduction	TCID_50_ assay	[35]
Bovine coronavirus (Kakegawa)	Enveloped ss-RNA virus/animal pathogen	Virus suspension	1:9, 2 s	4-log reduction	TCID_50_ assay	[35]
Swine influenza A virus (A/Swine/Ibaraki/46/2010 (H1N1))	Enveloped ss-RNA virus/animal pathogen	Virus suspension	1:9, 10 s	4-log reduction	TCID_50_ assay	[35]
Equine influenza virus (A/Equine/Hayakita/1/2007 (H3N8))	Enveloped ss-RNA virus/animal pathogen	Virus suspension	1:9, 30 s	3-log reduction	TCID_50_ assay	[35]
Bovine viral diarrhea virus I (Nose)	Enveloped ss-RNA virus/animal pathogen	Virus suspension	1:9, 30 min	4-log reduction	TCID_50_ assay	[35]
Bovine viral diarrhea virus II (KZ-91CP)	Enveloped ss-RNA virus/animal pathogen	Virus suspension	1:9, 30 min	4-log reduction	TCID_50_ assay	[35]
Foot-and-mouth disease virus (type A)	Non-enveloped ss-RNA virus/animal pathogen	Virus suspension	1:9, 60 min	4-log reduction	TCID_50_ assay	[35]
Foot-and-mouth disease virus (type O)	Non-enveloped ss-RNA virus/animal pathogen	Virus suspension	1:9, 60 min	3-log reduction	TCID_50_ assay	[35]
Foot-and-mouth disease virus (type Asia 1)	Non-enveloped ss-RNA virus/animal pathogen	Virus suspension	1:9, 60 min	4-log reduction	TCID_50_ assay	[35]
Bovine rhinitis B virus (EC11)	Non-enveloped ss-RNA virus/animal pathogen	Virus suspension	1:9, 2 s	4-log reduction	TCID_50_ assay	[35]
Feline calicivirus (F9)	Non-enveloped ss-RNA virus/animal pathogen	Virus suspension	1:9, 2 s	4-log reduction	TCID_50_ assay	[35]
Bovine rotavirus (22R)	Non-enveloped ds-RNA virus/animal pathogen	Virus suspension	1:9, 2 s	4-log reduction	TCID_50_ assay	[35]
Bulbul orthoreovirus (Pycno-1)	Non-enveloped ds-RNA virus/animal pathogen	Virus suspension	1:9, 2 s	3-log reduction	TCID_50_ assay	[35]

ds-DNA: double-stranded DNA; ss-DNA: single-stranded DNA; ds-RNA: double-stranded RNA; ss-RNA: single-stranded RNA; PMA: propidium monoazide; RT-qPCR: quantitative reverse transcription polymerase chain reaction; TCID_50_: 50% tissue culture infectious dose. For clarity, decimal places in the numbers of log_10_ reduction have been omitted.

**Table 3:** Inactivation of prions by CAC-717.

Microorganism (Strain or Type)	Classification	Sample Type	Reaction Volume Ratio (Sample: CAC-717), Exposure Time	Inactivation Effect	Evaluation Method	References
Prion (Chandler)	Scrapie prion/animal pathogen	Prion-infected cell lysate (ScN2a)	1:1, 1 h	4-log reduction	PMCA	[58]
Prion (Chandler)	Scrapie prion/animal pathogen	Brain homogenate	30% (*w*/*v*) of CAC-717 ceramic, 30 min	3-log reduction	PMCA	[59]
Prion (Chandler)	Scrapie prion/animal pathogen	Brain homogenate	30% (*w*/*v*) of CAC-717 ceramic containing 4% SDS, 30 min	6-log reduction	PMCA	[59]
Prion (Chandler)	Scrapie prion/animal pathogen	Brain homogenate	double treatment with 30% (*w*/*v*) of CAC-717 ceramic, 30 min X 2	4-log reduction	PMCA	[59]

PMCA: protein misfolding cyclic amplification; SDS: sodium dodecyl sulfate. For clarity, decimal places in the numbers of log_10_ reduction have been omitted.

## Data Availability

The original contributions presented in this study are included in the article. Further inquiries can be directed to the corresponding author.
